# Assessment of Predictors of Difficult Laparoscopic Cholecystectomy by Clinico-Radiological Parameters at a Tertiary Hospital in Eastern India

**DOI:** 10.7759/cureus.72512

**Published:** 2024-10-27

**Authors:** Samir Toppo, Kumar Gaurav, Kamlesh Kumar, Krishan Kumar, Sanjana Verma, Sunil T Tudoo, Muklesh K Mehta, Praveenkumar A

**Affiliations:** 1 General Surgery, Rajendra Institute of Medical Sciences, Ranchi, IND

**Keywords:** biochemical, cholelithiasis, clinical, conversion, gallbladder, laparoscopic cholecystectomy, ultrasonography

## Abstract

Background: Laparoscopic cholecystectomy has become the standard treatment for gallbladder (GB) stones, favored for its minimally invasive approach. Despite its benefits, the procedure sometimes requires conversion to open cholecystectomy due to intra-operative challenges, with conversion rates varying between 1% and 13%. There are various preoperative predictors that help in identifying such difficult cases and help to proceed safely. This study aims to identify the preoperative factors that could predict the difficulty of laparoscopic cholecystectomy, thus anticipating the need for conversion to open surgery.

Methods: A prospective observational study was conducted at RIMS Ranchi, India, from May 2023 to May 2024, including a total of 93 patients with gallstone disease who underwent laparoscopic cholecystectomy. Clinical history including age, gender, presence of acute cholecystitis, previous attacks, and previous upper abdominal surgery; biochemical markers including white blood cell (WBC) count, total bilirubin and alkaline phosphatase (ALP), and ultrasonographic findings such as GB wall thickness, stone impacted at the neck of GB, contracted or distended GB, presence of pericholecystic fluid collection, Mirizzi’s syndrome and others were analyzed to identify predictors of conversion.

Results: Of the 93 patients included in our study, there were 28 males and 65 females with a ratio of 1:2.3. The age group varied from 14 to 72 years with conversion to open cholecystectomy seen between the age group of 31-70 (mean age 49 years). We observed that 10 patients (conversion rate of 10.75%) underwent conversion from laparoscopic to open cholecystectomy. Significant predictors included acute cholecystitis, multiple previous attacks, and ultrasonographic findings of contracted GB.

Conclusion: Preoperative identification of patients at higher risk for conversion can enhance surgical planning and patient counseling, potentially improving outcomes in laparoscopic cholecystectomy.

## Introduction

Laparoscopic cholecystectomy (LC) is the treatment of choice for the majority of patients with gallbladder (GB) stones or cholecystitis. Among biliary tree procedures, it is the most common elective operation. There are many benefits to laparoscopic operations over open surgery. These include a shorter recovery time, less discomfort after the procedure, easier oral intake, less time spent in the hospital, and improvement in cosmetic results [1].

However, conversion to an open procedure is necessary for 1%-13% of LCs. The levels of difficulty are often challenging to predict [2]. It is therefore important to counsel the patient on the likelihood of conversion to open surgery. Surgeons can also improve postoperative results by mentally preparing for the difficult cholecystectomy. This preparation includes having an experienced surgical team, creating a thorough plan, performing an intraoperative cholangiogram, and overall being prepared.

Conversion from a laparoscopic to open surgery is mainly determined by the surgeon's experience, the patient's health, and technical considerations. Conversion is mostly initiated by problems with dissection and the need to view the anatomy properly. As for acute cholecystitis, the conversion rate can rise to 30%, while for elective LC, it is usually around 5% [2,3]. Although a consensus on what attributes specifically make LC difficult has not been reached, some studies have reported several major risk factors. These are the presence of dense adhesions at the frozen triangle of Calot's, which cannot be treated laparoscopically safely; a contracted and fibrotic GB; previous surgeries in the upper abdomen; a gangrenous GB; acute inflammation; and an emphysematous GB, and other conditions such as Mirizzi's syndrome. Other contributors to this include a previous cholecystostomy and the presence of a cholecystoduodenal or gastric fistula [2-5].

Knowing the predictions for completing such difficult operations is desirable. To predict the possibility of problems and the necessity of open surgery, one can look at patient demographics (age, gender, weight, co-morbidity, prior abdominal surgery, American Society of Anesthesiologists (ASA) score), clinical findings (acute versus chronic cholecystitis), and the experience of the surgeon, among other things [6-8].

When it comes to screening for cholecystitis and cholelithiasis, ultrasonography (USG) is by far the most popular, dependable, noninvasive, safe, and extremely accurate option. Surgeons can also use it to gain a better understanding of the patient's anatomy and any potential complications that may arise during surgery. USG not only helps with diagnosis but can also assist in assessing the complexity of the surgery by identifying anatomical challenges. A difficulty to grasp contracted fibrotic GB is indicated by an essential ultrasonographic finding, a maximal GB wall thickness of >4 mm. In addition to this, USG can show a porcelain GB, and GB containing a large, impacted stone, particularly in Hartmann's pouch. LC becomes more challenging in cases with pericholecystic collection, which indicates inflammation surrounding the GB [2-9].

Aims and objectives 

The aim is to assess predictors of difficult LCs using clinical, biochemical, and radiological parameters and to calculate the conversion rate.

## Materials and methods

This prospective observational study took place at the Department of General Surgery, Rajendra Institute of Medical Sciences (RIMS), Ranchi, India, from May 2023 to May 2024, after approval from the Institutional Ethics Committee of RIMS, Ranchi (MEMO No. 148, dated July 24, 2023). Using the formula S=z2*p(1-p)/e2 (S is the sample size, z is the degree of confidence, p is the proportion, and e is the margin of error), the sample size was calculated with z = 1.96, with a 95% confidence interval (CI), p = 0.3 and e = 0.1, which came to be S = 84. The patients recruited in our study were 93 [10].

All 93 consecutive patients who were admitted for LC within the trial period who had GB disease with ASA-Physical Status (ASA-PS) score I/II were included in the study. Exclusion criteria included patients with the presence of common bile duct stones, an episode of acute cholecystitis of less than 72 hours, and LC combined with other surgeries.

**Figure 1:**
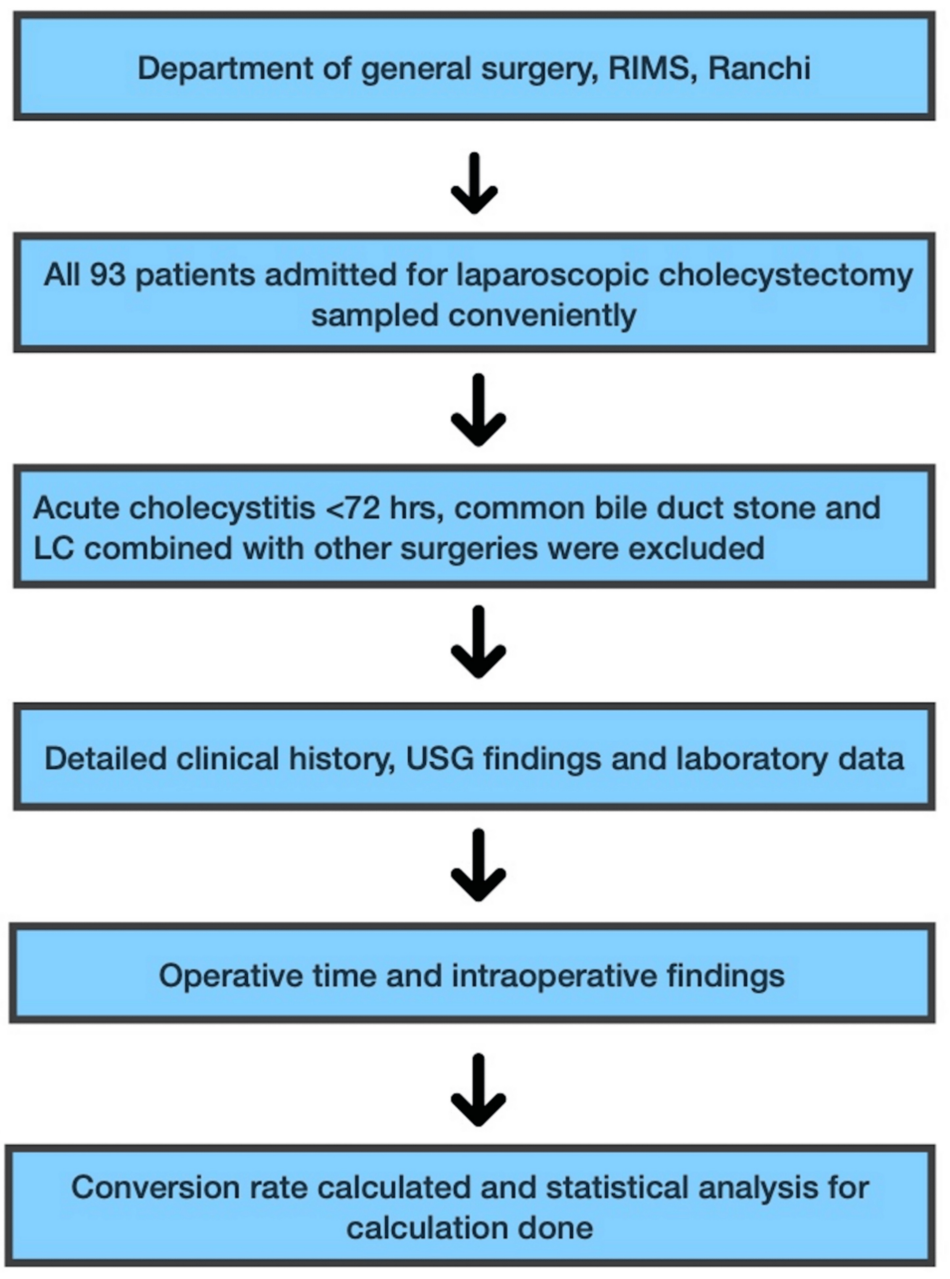
A flowchart of the study's overall methodology RIMS: Rajendra Institute of Medical Sciences, LC: Laparoscopic cholecystectomy

A complete history of all patients was taken followed by a complete physical examination and investigations including ultrasound of the whole abdomen and pelvis, liver function test, and complete blood counts were noted. Important clinical history noted were age, gender, presence of acute cholecystitis, previous attacks, and previous upper abdominal surgery. In order to identify potential complications during LC, all patients received abdominal and pelvic USG after an overnight fast. GB wall thickness, stone impacted at the neck of GB, contracted or distended GB, presence of pericholecystic fluid collection, Mirizzi syndrome, and others, like common bile duct size and gangrenous GB were noted. All patients underwent routine blood investigations and important parameters that may predict difficult LC including white blood cell (WBC) count, total bilirubin, and alkaline phosphatase (ALP) were noted. Instances that were deemed problematic were those involving injuries to the cystic duct or cystic artery, duration of surgery, or instances that required conversion to open cholecystectomy. A flowchart summarizing the research's methodology is shown in Figure 1.

Statistical analysis

The data were first entered into a Microsoft Excel spreadsheet (student version 2021; Microsoft, Washington, DC, USA) and then transferred to SPSS 27.0 (IBM Corp., Armonk, NY, USA) for statistical analysis. The statistical technique employed was the unpaired t-test, and the continuous variables were reported as mean ± SD. This included age. Frequencies and percentages were used to summarize categorical variables. They included age, gender, presence of acute attack, previous attacks, previous upper abdominal surgery, GB wall thickness, stone impacted at the neck of GB, contracted or distended GB, presence of pericholecystic fluid collection, Mirizzi syndrome, WBC count, total bilirubin and ALP. We utilized a chi-square test to examine the results. A significant p-value was defined as less than 0.05.

## Results

**Figure 2:**
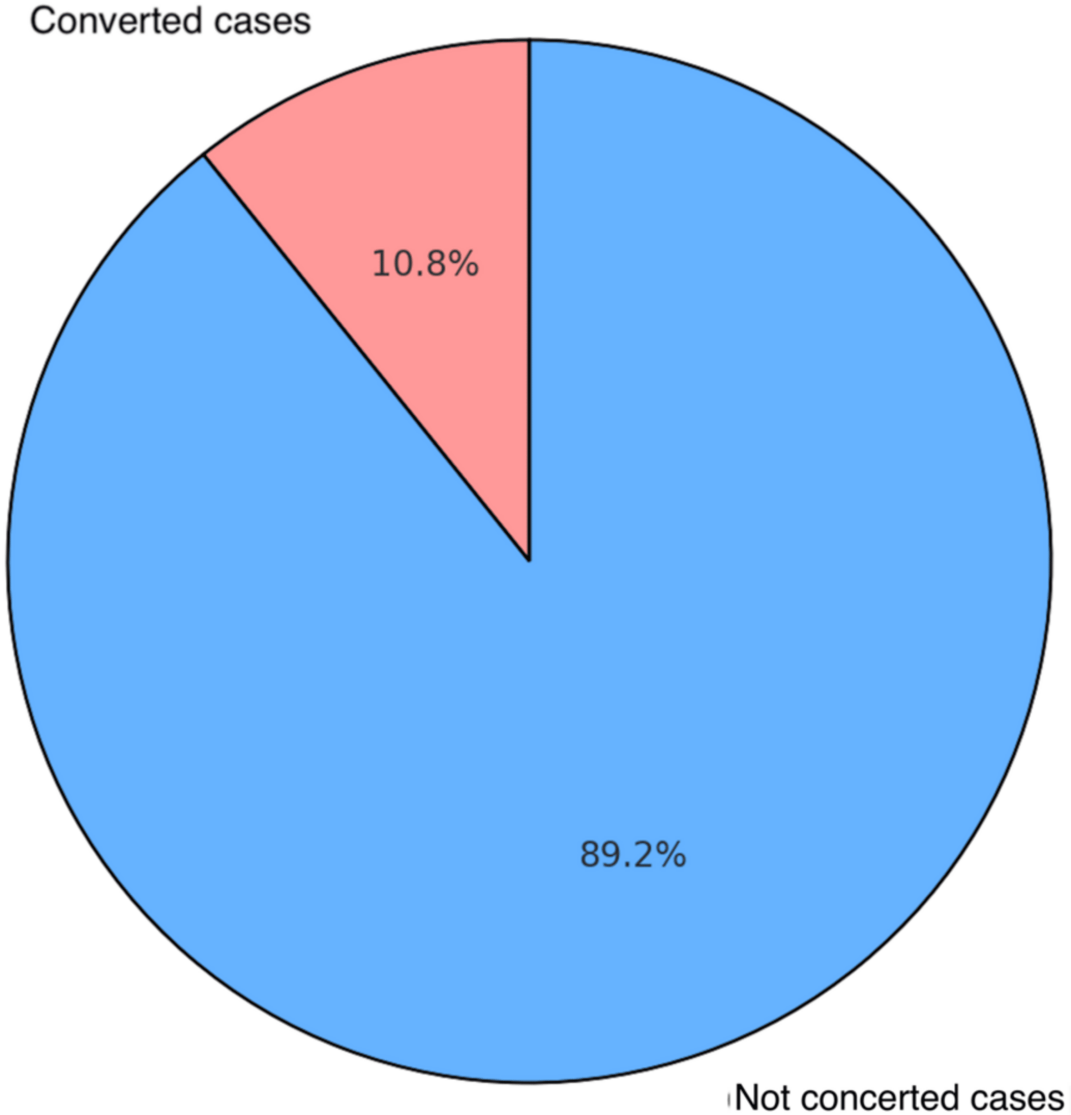
Total number of patients converted to open cholecystectomy

For this research, a total of 93 patients were chosen from among those who underwent LC due to gallstone illness. Preoperative clinical, biochemical, and USG data classified all cases as easy or challenging for LC. Both before and during the procedure, the reasons for the need to do an open cholecystectomy were investigated. Overall, 10 patients required a conversion to open cholecystectomy. The rate of conversion was 10.75% (Figure 2).

**Figure 3:**
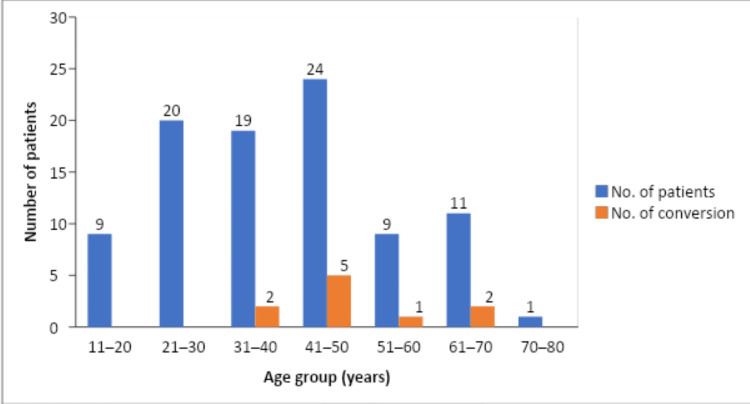
Conversion to open cholecystectomy among different age groups

The age distribution varied from 14 to 72 years with the majority of patients who underwent conversion from laparoscopic to open cholecystectomy falling in the age group of 31-70 years. The mean age in the conversion group was 49 years with a standard deviation of 12.02 (Figure 3).

**Figure 4:**
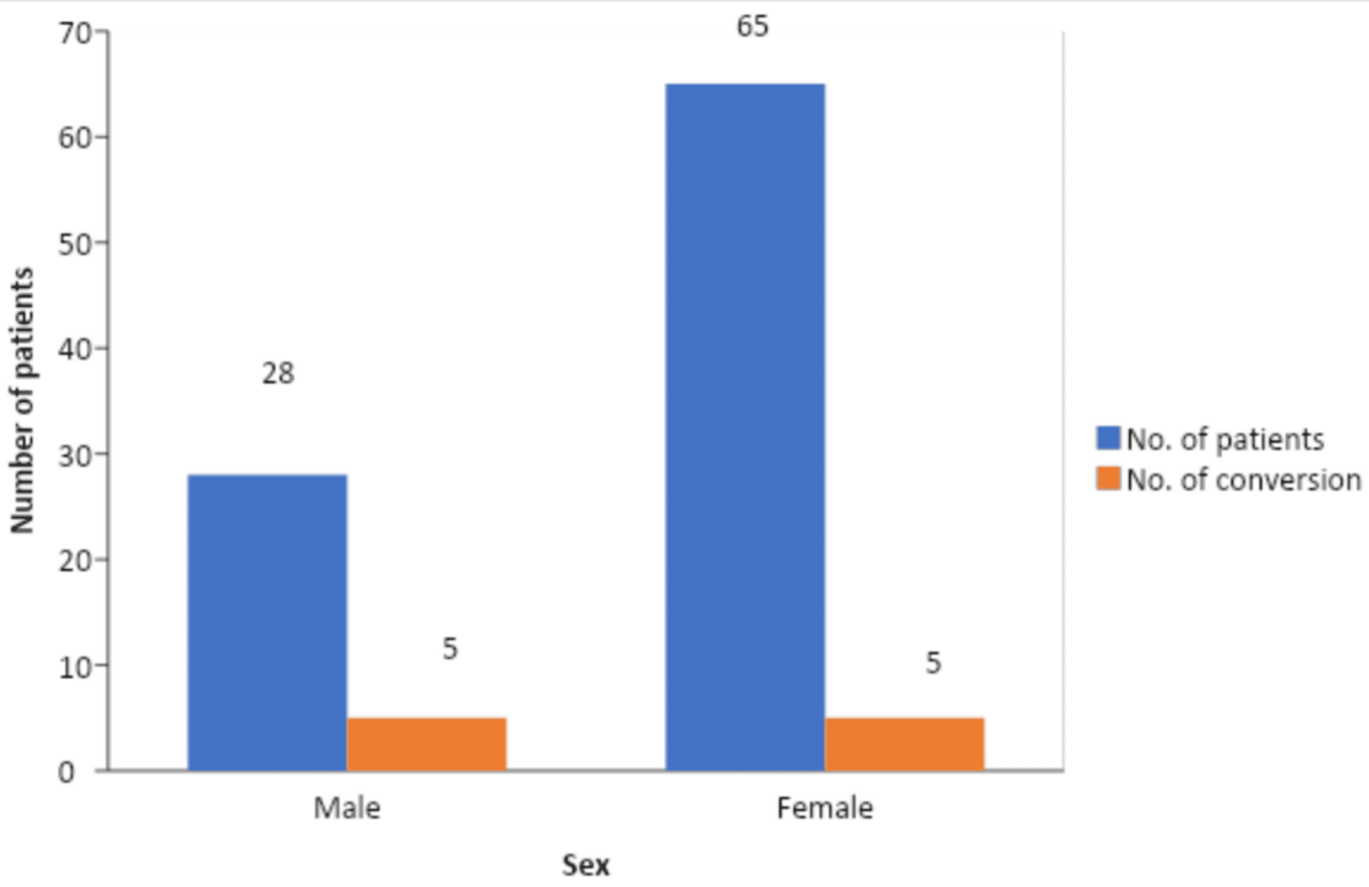
Conversion to open cholecystectomy among genders

The male-to-female ratio in our study population was 1:2.3. The number of female patients in our study was higher, and 7.69% of all female patients underwent conversion to open cholecystectomy, in contrast, 17.85% of all the male patients who underwent conversion to open cholecystectomy (Figure 4).

**Figure 5:**
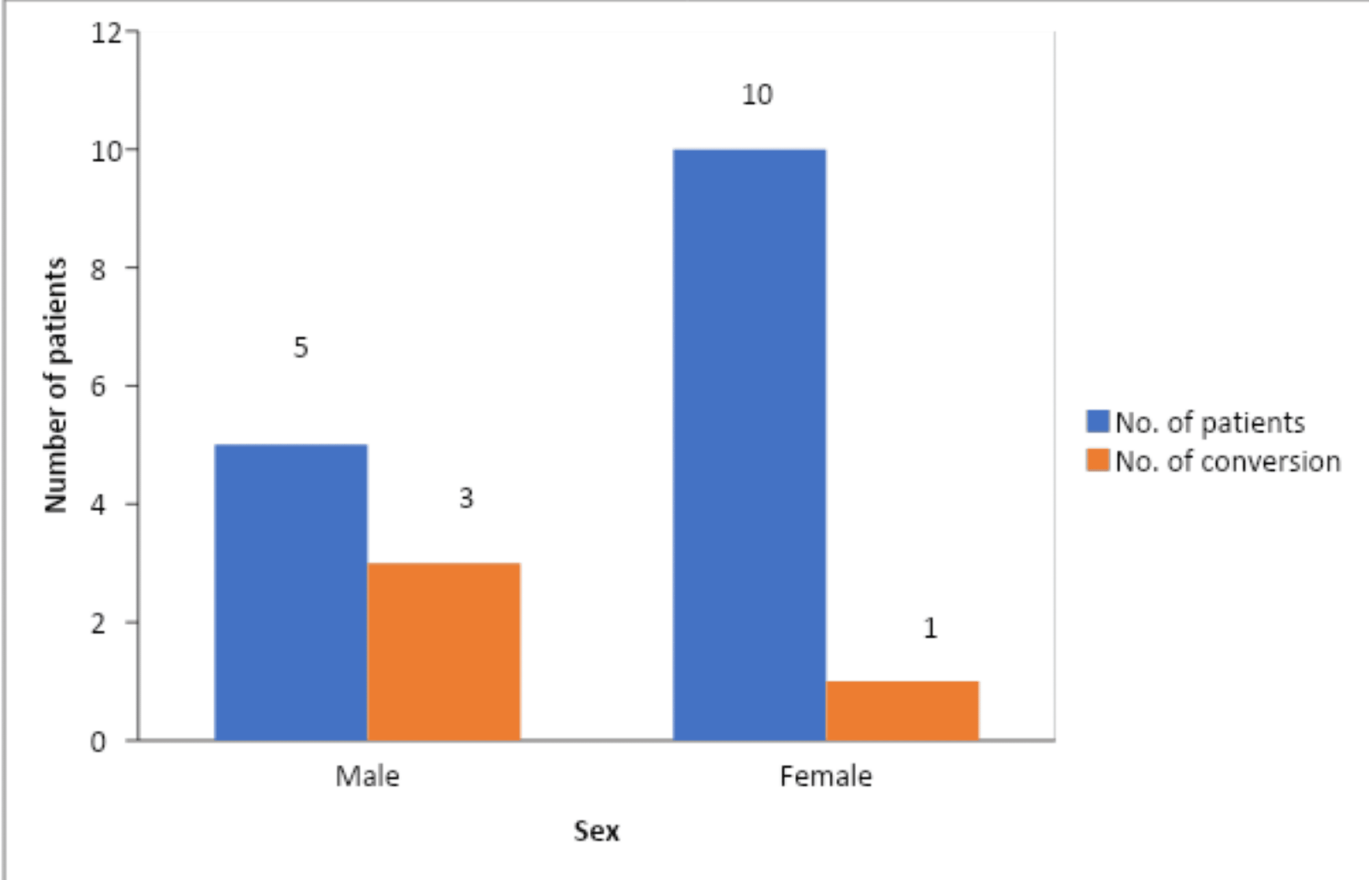
Conversion to open cholecystectomy among genders in setting of acute cholecystitis

Acute cholecystitis was present in 15 patients in our study, out of which 26.67% (n=4) needed conversion to open cholecystectomy (Figure 5).

**Figure 6:**
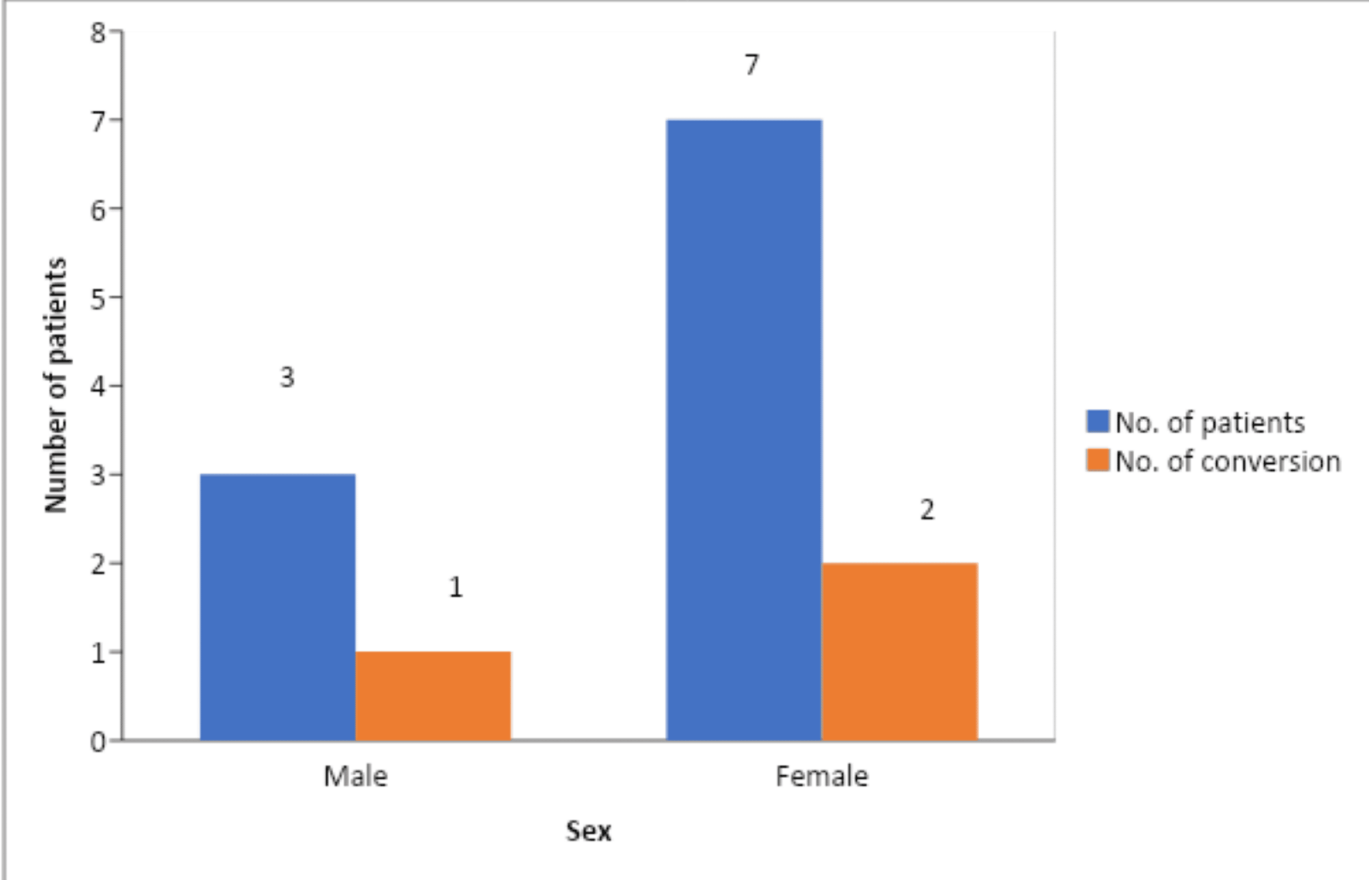
Conversion to open cholecystectomy among genders who had ≥2 attacks of cholecystitis

Out of the 10 patients who presented with two or more episodes of cholecystitis, 30% (n=3) of the patients converted to open cholecystectomy (Figure 6).

**Figure 7:**
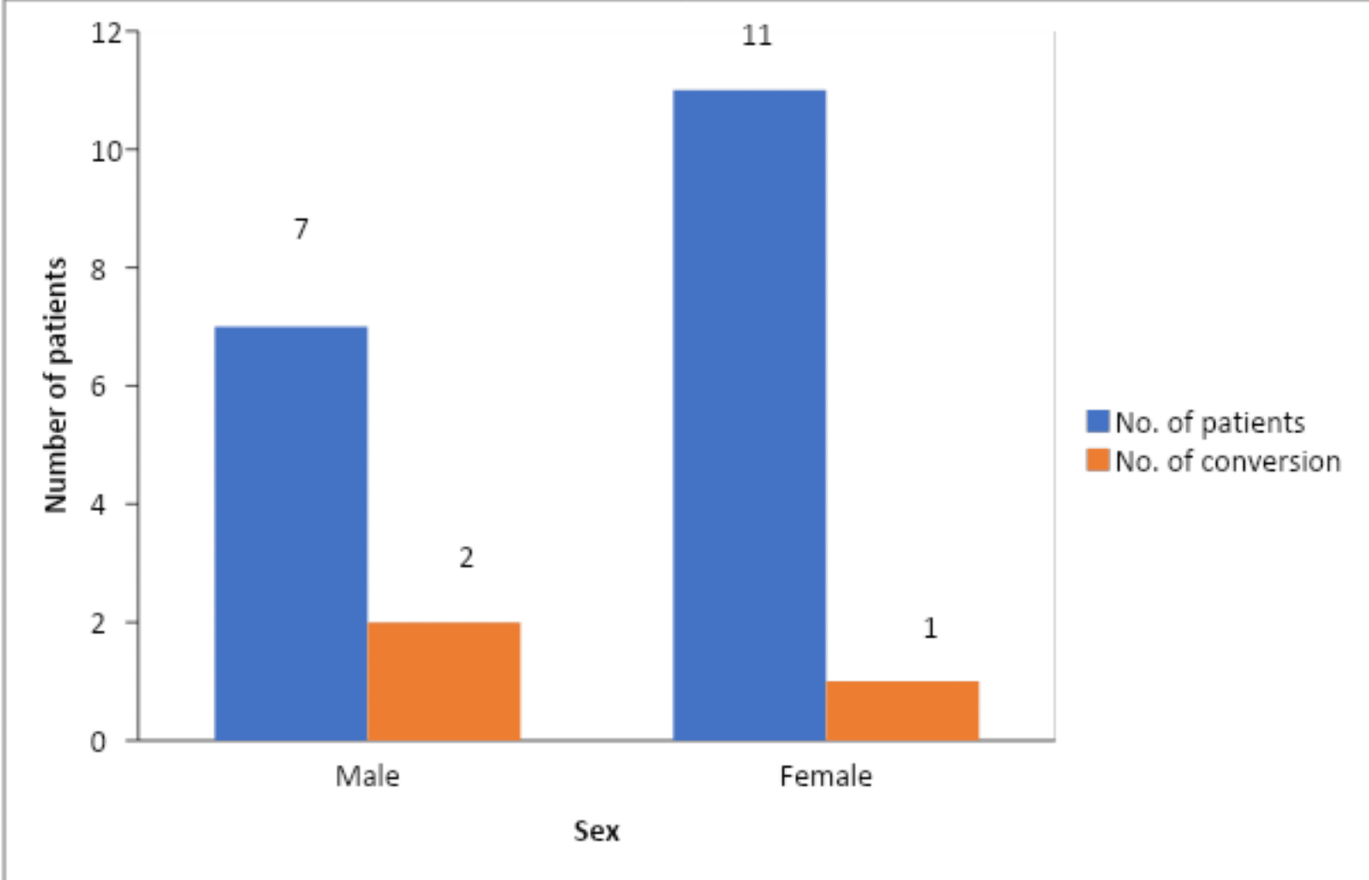
Conversion to open cholecystectomy among genders in patients who had GB wall thickness ≥4 mm

There was no conversion seen in the three patients presenting with a history of upper abdominal surgery. 14% (n=1) of total patients with raised WBC count >10,000 cu/mm and 9% (n=1) of total patients with raised total bilirubin >1.2 mg/dl got converted to open cholecystectomy. Among the patients with GB wall thickness ≥ 4mm (n=18), 16.67% (n=3) were converted to open cholecystectomy (Figure 7).

**Figure 8:**
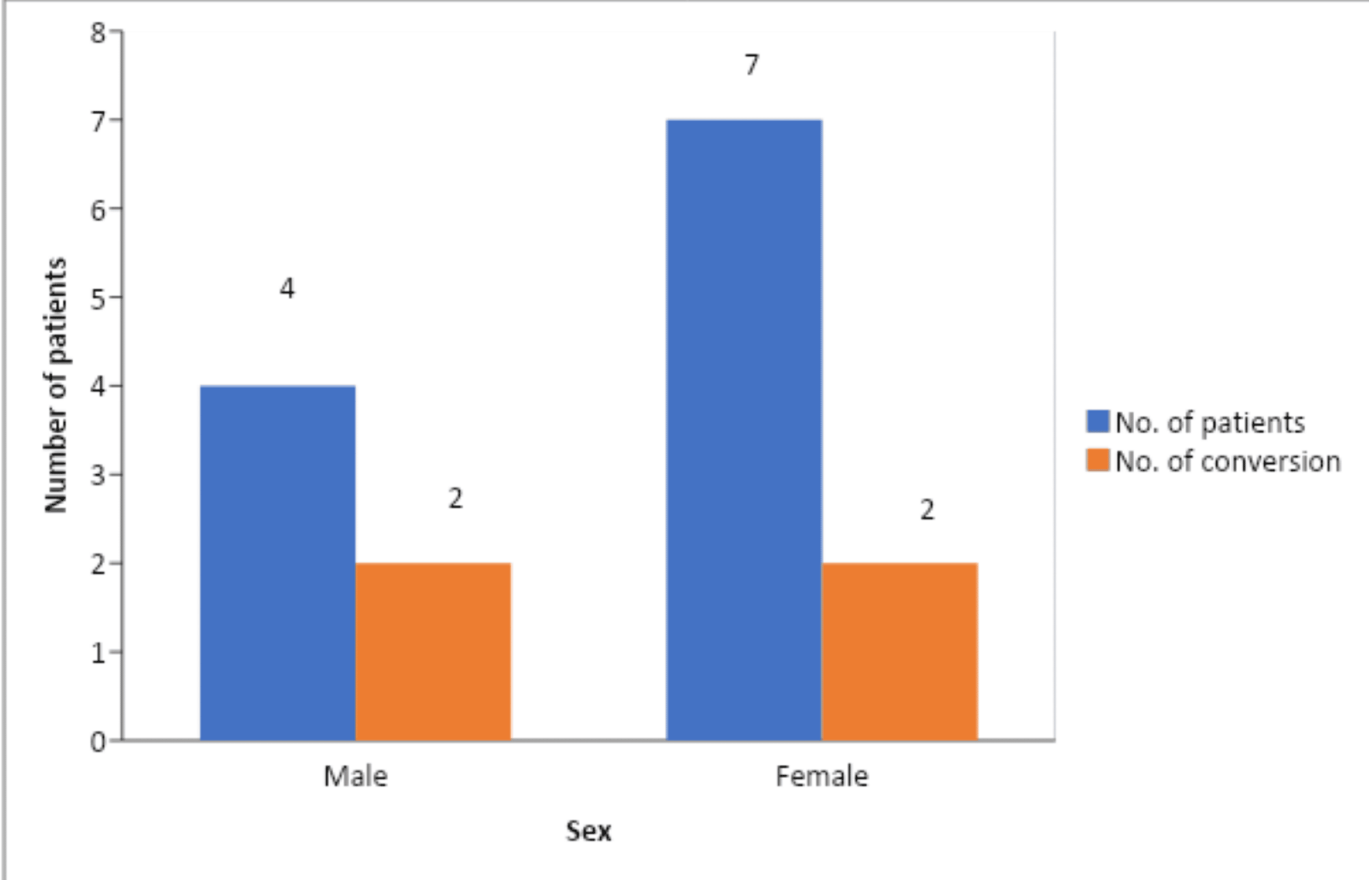
Conversion to open cholecystectomy among genders in patients with contracted GB

Among the 11 patients who contracted GB encountered on USG, 36.36% (n=4) got converted to open cholecystectomy (Figure 8).

Among the three patients with stone impacted at the neck/cystic duct, 33.34% (n=1) needed conversion to open cholecystectomy. There were seven patients with the presence of pericholecystic fluid collection, of which 28% (n=2) and 100% (n=1) of patients diagnosed with Mirizzi’s syndrome underwent conversion from laparoscopic to open cholecystectomy. In the per-operative causes of conversion to open cholecystectomy, dense adhesions causing difficulty in identifying the critical view of safety was the most important factor, followed by contracted GB (Table 1).

**Table 1:** Per-operative causes of conversion

Per-operative causes of conversion	No. of conversions (%)
Dense adhesions and frozen Calot’s triangle	6 (6.45)
Anatomic variation of cystic duct	1 (1.07)
Oedematous gall bladder wall with adhesion	1 (1.07)
Contracted GB	2 (2.15)
Total cases converted	10 (10.75)

Among the clinical parameters (age, gender, presence of acute cholecystitis, previous attacks of acute cholecystitis, and previous upper abdominal surgery), 15 cases were predicted to be difficult, out of which nine were deemed challenging intra-operatively, and three had to be converted into open cholecystectomy. Among the biochemical parameters (WBC, total bilirubin, and ALP), 11 cases were predicted to be difficult, of which seven were deemed difficult intra-operatively and two cases were converted to open. With the USG findings, 20 cases were predicted to be difficult, 13 cases were found to be difficult intra-operatively and five cases had to be converted to open. On statistical analysis, comparison between variables and conversion rate, patients presenting with acute attack, two or more attacks of acute cholecystitis, and contracted GB were statistically significant (Table 2).

**Table 2:** Statistical analysis showing different variables and their significance Chi-square test was used to analyze the relationship between conversion of cholecystectomy and the predicting variables. NS: Non-significant, S: Significant, WBC: White blood cell, ALP: Alkaline phosphatase, GB: Gallbladder

Preoperative parameters	Predicted as difficult	Concluded as difficult	Conversion	P-value
Age (≥65 years)	9	5	2	0.24 (NS)
Sex (male)	28	13	5	0.14 (NS)
Acute cholecystitis	15	9	4	0.03 (S)
No of acute attack (≥2)	10	6	3	0.03 (S)
History of upper abdominal surgery	3	3	0	0.54 (NS)
WBC (≥ 10,000/cumm)	7	3	1	0.75 (NS)
Total bilirubin (>1.2 mg/dL)	11	4	1	0.84 (NS)
ALP (>310 IU/L)	1	1	0	0.72 (NS)
Pericholecystic collection	7	7	2	0.11 (NS)
GB wall thickness (≥4mm)	18	12	3	0.36 (NS)
Stone impaction at neck	3	1	1	0.19 (NS)
Mirizzi’s syndrome	1	1	1	0.07 (NS)
GB contracted	11	9	4	0.003 (S)

## Discussion

Indeed, gallstone disease is on the rise affecting a fair number of people, and cholecystectomy is currently the most effective treatment for gallstone disease, surpassing appendicectomy as the most frequently performed surgery in the UK (Maingot 1774) [1]. A traditional open cholecystectomy is performed through, either the sub-costal Kocher’s incision or the right para-median incision. Recovery periods, scars, and postoperative pain are significant with standard open cholecystectomy compared to LC. The field of laparoscopy has made remarkable strides in the past decade. LC is the procedure of choice for the treatment of symptomatic gallstone disease. Ranchi's Rajendra Institute of Medical Sciences ranks high among the procedures performed most often. Finding out what factors influence surgeons' decisions to have open cholecystectomy versus laparoscopic is the major motivation for this study.

Patients with severe adhesions and malformed anatomy may present with difficulties during LC. Acute cholecystitis, twisted anatomy, an empyema or contracted GB, Mirizzi's syndrome, previous upper abdominal surgery, and adhesions in the Calot's triangle are just a few of the many factors that might make the process more technically demanding. Conversion rates have been found to vary between 1.5% and 35% across several studies. This study examined the ultrasonographic, biochemical, and clinical indicators to forecast difficult LC in 93 patients [11-16].

Factors that have been found in the literature to be predictive of a difficult LC include age, gender, acute cholecystitis at presentation, previous surgery on the upper abdomen, obesity, high WBC count, low albumin, gallstone size, GB wall thickness, number of stones, common bile duct diameter, and impacted stone at GB neck. Of these, patients older than 65 years old, a history of upper abdominal surgery, a thick GB wall, a constricted GB, an impacted stone at GB neck, and male sex showed maximum correlation [2-15].

Open cholecystectomy was performed in 10 out of 93 cases (or 10.75%) in this study. Multiple additional research has also shown similar conversion rates. The policy of early conversion of difficult laparoscopic cases in our institute explains the conversation rate to be on the higher end. According to Sharma et al., the conversion rate was 8.8%, which most commonly occurred due to the inability to identify the calot's triangle anatomy [11-17].

The patients' ages varied from 14 to 72 years and conversion to open cholecystectomy was observed in the age bracket of 31 to 70 years. Because most patients admitted to our hospital were of this age group, this is also the age group that showed the highest conversion rate. Only two of the 93 patients admitted were over the age of 65 and subsequently converted. It has been observed that the likelihood of conversion increases as one ages. However, in our study, there was no significant relationship between age and conversion. The small number of participants aged 65 and above may account for the disparity. Using multivariate logistic regression analysis, van der Steeg et al. discovered that age above 65 was a significant independent predictor for conversion [18].

Among the 93 participants, 65 were female and 28 were male. The results of our study, however, showed that conversion was unaffected by gender. The fact that there were more women, owing to the higher prevalence of gallstones in them, in our study might account for this. However, previous research by Volcan et al. and van der Steeg et al. found that the conversion rate was twice as high in male patients (2011) [18,19].

Out of 15 patients who presented with an acute onset of cholecystitis, four patients required conversion to open cholecystectomy. In case of acute cholecystitis, the GB wall is friable and edematous which makes it difficult to hold the fundus. In addition, adhesions around the Calot’s make dissection difficult. Being statistically significant, this result agrees with that of the study by van der Steeg et al., which found that conversion could be strongly predicted by acute cholecystitis [18].

A history of more than two attacks was found in 10 patients, three of those patients required conversion. With repeated attacks of cholecystitis, the GB wall thickens, and more adhesions occur causing difficulty in dissection. Consistent with the work of Li et al., we discovered that the likelihood of conversion increased significantly when the number of acute attacks exceeded two [20].

This analysis did not find a significant relationship between prior upper abdominal surgery and conversion to open cholecystectomy. On the other hand, Fanaei et al. discovered that a history of upper abdominal surgery strongly indicated prognosis [21]. That difference might be explained by the small number of patients who had previous upper abdominal surgery.

Out of the 93 patients, 15 were identified as potentially challenging cases based on clinical criteria, nine of these experienced complications during surgery, and three cases were converted to open cholecystectomy. Among the clinical indicators, acute cholecystitis and bouts of cholecystitis greater than or equal to two were significant predictors. This agrees with research done by van der Steeg et al. and Li et al. [18-20].

The biochemical parameters including raised WBC count, elevated total bilirubin, and ALP are significant predictors of difficult LC in studies done by Lipman et al., Li et al., and Gholipour et al. [20,22,23]. Although our study did not find such a correlation. This disparity is likely explained by the high number of patients presenting in our institute who belong to the low socioeconomic group and who present late in the course of disease and require stabilization prior to surgery. Further, most of our patients' biochemical markers were within normal limits prior to surgery and considered routine cases.

Multiple attacks and chronic cholecystitis make the GB fibrosis and contract, making the dissection difficult. Consistent with a study by Iqbal et al. that discovered contracted GB and Mirizzi’s syndrome to be crucial, our investigation also demonstrated the same. Contrary to what Carmody et al. found, our research did not find an association between the GB wall thickness and the decision to convert to open cholecystectomy. Several literature show that there is a positive correlation between GB wall thickness and conversion to the open method, as it makes grasping and manipulation of the GB difficult [24-30].

In contrast to previous research which found a moderate link between stone impaction and the need for an open operation to remove gallstones from the GB neck, our study found no such relationship [28,29]. The main problem with a neck-impacted stone, also known as Hartman's pouch, is that it can lead to the formation of a mucocele, which makes holding the GB during dissection much more difficult. In numerous cases in our study, aspirating the GB's contents helped to empty it, making it easier to manage. Skillful handling of challenging situations has also resulted from an expansion in surgeons' knowledge and improvements in laparoscopic procedures over the past decade. Our study included five patients whose USG revealed pericholecystic fluid; two of these patients underwent conversion to open cholecystectomy as a result.

Adhesions made dissection a challenge during LC, which led to more complications and eventually mandating an open procedure. Other reasons for conversion include GB tears leading to the leakage of stones and bile, bleeding from the GB bed and cystic artery rip, and the inability to apply clips because of cystic duct anatomical abnormalities. Cases that were deemed difficult included a variety of challenges that led to an operating duration exceeding 90 minutes. A LC, when performed by an experienced surgeon, should not take more than 45 to 50 minutes [28,29].

Difficult LC can be reasonably well predicted by preoperative clinical, biochemical, and ultrasonographic variables, according to this study. These tests can also reveal when an open cholecystectomy is the best option and when the patient needs to be appropriately counseled about the likelihood of conversion and outcomes of an open procedure, including longer hospital stays and more extensive postoperative management, before undertaking a challenging case.

The limitation of this study was that it was limited to a single hospital-based study, a very small cohort was selected, and therefore, cannot be used as a representation of a larger population. Many patients in our study came from a lower socioeconomic background and presented late in an acute stage requiring stabilization prior to an interval cholecystectomy; therefore, many acute cases could not be included in this study. Additionally, there could be bias related to the surgeons because many surgeons these days choose and are adept in LC.

## Conclusions

The presence of acute cholecystitis, a greater number of previous attacks, and contracted GB are significant predictors of difficult cases of LC. Both the patient and the surgical team gain a lot when challenging LC situations are identified before surgery. A conversion to open cholecystectomy should not be seen as a flaw on the surgeon's part, but rather a necessary precaution to prevent major complications. However, there is a need for more randomized controlled studies and multivariate analysis to evaluate the efficacy of various predictors to plan an appropriate procedure for efficient patient care.
